# Bisphenol S Adsorption Behavior on Ferralsol and Biochar Modified Soil with Dissolved Organic Matter

**DOI:** 10.3390/ijerph16050764

**Published:** 2019-03-03

**Authors:** Shiqiu Zhang, Xue Yang, Le Liu, Kui Zheng, Meiting Ju, Jinpeng Liu

**Affiliations:** 1 College of Environmental Science and Engineering, Nankai University, 38 Tongyan Road, Tianjin 300350, China; swustzsq@sina.com (S.Z.); yx212017@sohu.com (X.Y.); huanjikejian@sohu.com (L.L.); 2 Analytical and Testing Center, Southwest University of Science and Technology, Mianyang 621010, China; zhengkui@swust.edu.cn

**Keywords:** DOM, biochar, Ferralsol, BPs, adsorption

## Abstract

Bisphenol S (BPs) has been found in a variety of common consumer products surrounding human living, despite the fact that it could damage the human digestive system and genital system. In China, straw-returning to the field is a common soil improvement technology used to increase the concentration of dissolved organic matter (DOM), which plays an important role in the natural environment as a microreactor of contaminants. Additionally, the biochar obtained by the straw is an effective soil conditioner. DOM is a key influencing factor when biochar is employed as the conditioner of BPs contaminated soil. However, the BPs adsorption behavior on the Ferralsol affected by DOM and biochar is also unclear. Hence, DOM was prepared and the effect of DOM on the BPs adsorption behavior on soil and biochar modified soil was investigated. DOM was characterized by Elemental analysis, Fourier transforming infrared spectra (FT-IR), and three-dimensional excitation-emission matrix spectra (3D-EEM). The results of the adsorption experiments indicated that both biochar and DOM could improve the BPs adsorption capacity in Ferralsol, while DOM suppressed the BPs adsorption capacity of biochar modified soil, indicating that DOM and BPs could not be applied at the same time for BPs adsorption.

## 1. Introduction

In recent years, as straw-returning to the field has become a popular strategy in China, the concentration of dissolved organic matter (DOM) has increased in the soil environment, and many chemical reactions will have occurred between contaminants and DOM, including complexation, redox reactions, and photodegradation [1,2]. DOM, a more mobile and bioavailable fraction of organic matter in the soil environment, is commonly defined as the organic matter remaining in-solution through 0.45-μm filtration [3]. Moreover, DOM plays an important role in the natural environment as a microreactor of contaminants which could enhance the reactivity of contaminants by bringing them into close association with a very reactive intermediate, and its important influences on the environmental behavior of contaminants have been widely reported [4,5]. However, the constituents of DOM are extremely complicated, caused by various sources and surrounding environments, including non-humus (such as saccharide, amino acid, protein, lignin, and organic acid) and humus (such as humic acid, fulvic acid, and humin) [6,7]. The molecular characterization is still elusive, and interaction between DOM and environmental matrices is also poorly understood.

Bisphenol A (BPa), an important chemical intermediate, is primarily used in the production of epoxy resins and polycarbonate plastics [8,9]. However, a number of countries have banned the usage of products suspected of containing BPa [10]. Many pieces of literature indicate that bisphenol S (4, 4′-sulfonyldiphenol, BPs, shown in Figure S1) is extensively applied in the replacement of BPa [11,12,13]. Additionally, BPs has already been documented as a contaminant in a variety of food products sold in the United States [14]. Similarly to BPA, BPs has been used in epoxy glues and polymerization reactions [15]. Driven by increasing awareness and demand, large amounts of BPs are exposed to humans, and it has been detected in urine samples [16,17]. Once BPs enters the human system, it not only has similar hormone-mimicking characteristics to BPa, but also appears to be more resistant to environmental degradation than BPa [18]. In addition, Patricia et al. reported that the BPs induces meiotic effects in both sexes that, in males, may persist for several generations [19]. Compared to BPa, BPs is more heat- and light-resistant, and it is more resistant to environmental degradation and may possess higher environmental resistance [20]. With the widespread usage of BPs, it may discharge into the soil environment through sewage irrigation [14,16]. The extent of BPs adsorption in soil will depend on the ionic strength or pH of the adsorption solution, as these parameters affect both the BPs solubility and available adsorption sites [21,22]. Red acid soil (RAS, Ferralsol) is one of the common soil types in southern China, mainly distributed in the low hilly areas south of the Yangtze River, including Jiangxi Province and Hunan Province. The red acid soil in the area belongs to the ferrasol soil. This area, as a critical part of the economy and agriculture, plays a significant role in the development of China, and the major crop in the region is rice paddy, with an annual output of 50 million tons [23,24]. Moreover, the use of biochar has attracted wide attention as an alternative method of soil remediation. Biochar is produced through the pyrolysis of biomass under limited oxygen supply, and the main raw materials of biochar are agricultural wastes (wood, straw, or shell), municipal solid wastes (refuse, sludge), and other organic materials [25]. Biochar has a high adsorption capacity due to its high surface area, aperture structure, high stability, higher cation exchange capacity, and high amounts of surface functional groups [26]. When biochar is employed as the conditioner of BPs contaminated soil, DOM is a key influencing factor. However, a lot of research focuses on the BPs adsorption capacities of biochar or soil [20,27], and studies on the interaction between the DOM and BPs adsorption of the soil and biochar modified soil are still lacking.

The object of this work is to explore the BPs adsorption behavior in Ferralsol and biochar modified Ferralsol (B-soil) with the impact of dissolved organic matter (DOM). The biochar sample was homemade using the rice paddy straw as the feedstock. The DOM sample was obtained from the decay of the paddy straw in the soil for a certain time and then extracted. The adsorption parameters included BPs and DOM adsorption on the samples, and the effect of DOM on the BPs adsorption behavior, adsorption kinetics, and adsorption thermodynamics. The evolution of DOM compositions was characterized by three-dimensional fluorescence spectroscopy (3D-EEM), FT-IR, and Element analysis.

## 2. Materials and Methods

### 2.1. Sample Preparation and Reagent

#### 2.1.1. Soil

The study area is mainly located in southern China, including Jiangxi Province and Hunan Province. The red acid soil (RAS) in the area belongs to the Ferralsol. The major crop in the region is rice paddy, with an annual output of 50 million tons. The soil sample was collected in the area (centered around 28°07′53.3″ N, 116°52′45.2″ E), with a surface area of 50 km^2^. The annual average temperature is about 17.6 °C, the annual average rainfall is about 1780 mm, and the average elevation is about 220 m. The bedrock type is sandstone. The soil sample was mixed. The sample was freeze-dried (FreeZone 6 Liter, Kansas City, MO, USA) for four days, and the sample was then sealed and stored in a glass bottle.

#### 2.1.2. Biochar

The preparation of biochar was as follows. The raw material used in the experiments was paddy straw. The sample was washed with deionized water and air-dried. Subsequently, the straw sample was turned into biochar via a slow anaerobic pyrolysis method at 500 °C for 2 h, with a heating rate of 4 K/min under a continuous N_2_ flow (50 mL·min^−1^). After pyrolysis, the biochar sample was passed through a 1-mm mesh size sieve and washed with deionized water to remove the DOM of biochar [28].

#### 2.1.3. B-soil

The B-soil sample was the biochar modified soil sample. According to the literature, a common amount of biochar added to soil is 4% [29,30]. Hence, 10 g of soil sample and 0.4 g of biochar sample were thoroughly mixed by mechanical stirring.

#### 2.1.4. DOM

As rice paddy was the main agricultural crop in the region, the rice paddy straw was the raw material employed for returning to the field. Firstly, the paddy straw was dried in the air and ground into fine particles through a 0.5-mm mesh size sieve. In order to obtain the DOM samples, 100 g of dried soil sample and 10 g of paddy straw were sufficiently mixed. The mixture was divided into 10 pieces, and each piece was added into the 250 mL conical flask with 4.8 mL deionized water. During the degradation, the temperature was controlled under 298 K and the water content was 40%, with regular water replenishment. Lastly, after the set time (0, 1, 5, 15, 30, 60, 90, 120 day), 100 mL deionized water was injected into the flask with vibrating for 2 h, and the filtrate was then filtered through the 0.45-μm membrane filter and freeze-dried until further use.

#### 2.1.5. Reagents

The water used in all the experiments was deionized water (18.25 MΩ·cm). Bisphenol S (BPs, Heowns Chemical Factory, Tianjin, China) was used in this study.

### 2.2. Physicochemical Analyses

**C/H/N/O/Ash:** The elemental carbon, hydrogen, and nitrogen contents of the soil, biochar, and DOM samples were measured by the Euro EA3000 Elemental Analyzer (Leeman, Hudson, NH, USA). The ash contents of the samples were tested by high-temperature roasting at 1073 K for 3 h under continuous air flow. The elemental O content was estimated by the mass difference (100%-C, H, N and ash %) [31]. The contents of C/H/N and ash compositions were reported using the average data executed in duplicate.

**SA:** For the surface areas (SA) analysis, the samples were determined by the ChemiSorb 2720 Analyzer (Micromeritics Instrument, Norcross, GA, USA) with N_2_ adsorption at 77 K. In addition, before N_2_ adsorption, the samples were outgassed at 105 °C for 16 h. The multipoint BET method was used for the surface area analysis [32].

**CEC:** For the cation exchange capacity (CEC), the saturating exchange sites of 1.0 g samples were determined with 40 mL of 1 mol·L^−1^ CH_3_COONH_4_ solution at pH 7, before then replacing the adsorbed NH_4_^+^ twice with 2 mol·L^−1^ KCl [33]. In between this step, the sample was washed by dimethylcarbinol. The mixture was shaken overnight prior to analysis. The NH_4_^+^ in KCl solution was tested using an ion chromatograph (ICS-5000, Dionex, Sunnyvale, CA, USA).

**FT-IR:** The FT-IR spectra were collected by the Spectrum One FT-IR spectrometer in the range of 4000–400 cm^−1^, with an average of 32 scans at resolution of 2 cm^−1^ (PerkinElmer, Waltham, MA, USA). The biochar sample and the KBr were mixed with the mass fraction of 1/10.

**3D-EEM:** The parameters for the 3D-EEM spectra obtained by the fluorescence spectrophotometer (FLS 920P) were as follows: PMT voltage was 700 V, excitation light was a 150 W xenon arc lamp, signal to noise ratio > 110, excitation wavelength range (Ex) was 200–550 nm, emission wavelength range (Em) was 250–6000 nm, the wavelength increment was set at 5 nm, and the scan speed was 2400 nm·min^−1^. DOM was diluted with deionized water until the TOC concentration was adjusted to 5 mg·L^−1^, and the deionized water was set as the blank control. The intensity of each area represents the relative DOM content, analyzing the change of components combined with fluorescence regional integration (FRI) [3,34]. The method could reduce the calculation time using the raw matrix without advanced mathematical effort. Volumes of Fluorescence (*F*) were calculated by the corrected matrix, following the integration method within each region (*i*):

(1) $$\Phi_{i}=MF\left(i\right)\iint I\left(\lambda_{ex}\lambda_{em}\right)d\lambda_{ex}d\lambda_{em}$$

where *MF*(*i*) was the multiplication factor, $d\lambda_{ex}$ was the excitation wavelength interval, $d\lambda_{em}$ was the emission wavelength interval, and $I\left(\lambda_{ex}\lambda_{em}\right)$ was the fluorescence intensity at each excitation-emission pair (Raman units).

### 2.3. Adsorption Experiments

The details of the batch experiments for the adsorption were as follows. All the adsorption experiments were executed using an air thermostatic shaker (HNYC-2102C, Honour Instrument, Tianjin), including the factors of DOM with different degrees of decay, contact times, initial BPs solution concentrations, and adsorption temperatures. (i) For the DOM and BPs adsorption, the appropriate solid DOM (120 days)/BPs, adsorbent (Soil/B-soil: 0.5000 g, Biochar: 0.0600 g), and 30.00 mL water were added into the 100 mL conical flask for 1440 min with a pH about 5.0–6.0. (ii) For the effect of DOM with different decay times, 30.00 mL 40.00 mg·L^−1^ BPs solution and adsorbent (Soil/B-soil: 0.5000 g, Biochar: 0.0600 g) were injected into the 100 mL conical flask for 1440 min with a pH of about 5.0–6.0. (iii) The adsorption kinetics was determined by analyzing the adsorption capacity at different time intervals (5–1440 min) with the same adsorbent dosage and initial BPs concentration in a 100 mL conical flask in which the pH was about 5.0–6.0. For adsorption isotherms, the different concentrations of BPs solution (20.00–80.00 mg·L^−1^, 20.00 mg·L^−1^ interval, 30.00 mL) were shook till the equilibrium achieved the same sorbent dosage in a 100 mL conical flask in which the pH was about 5.0–6.0. The temperature factor was investigated by determining the adsorption capacity at 298 K, 303 K, and 308 K. After injecting the DOM (120 days), the experiment repeated the above steps. Moreover, the DOM injected into the solution ensured that the DOC concentration was adjusted to 50.00 mg·L^−1^. In all the adsorption experiments, the mixtures were separated by a 0.45 μm filter membrane, and the BPs concentrations were measured at 258 nm with high-performance liquid chromatography (HPLC) equipped with a diode array detector (DAD).

### 2.4. Adsorption Kinetics Analysis

Two kinetic models were employed to investigate the mechanism of BPs adsorption on the biochar samples [35,36,37], including the pseudo-first-order kinetic model and the pseudo-second-order kinetic model.

The pseudo-first-order kinetic equation was as follows:

(2) $$\log\left(q_{e}-q_{t}\right)=\log q_{e}-\frac{k_{1}t}{2.303}$$

where, *k*_1_ was the equilibrium rate constant of the pseudo-first-order kinetic model (min^−1^) representing a quicker adsorption, and *q_e_* and *q_t_* were the BPs adsorption amount (mg·g^−1^) at equilibrium *t* and any time *t* (min) per unit weight of the adsorbent, respectively.

The pseudo-second-order kinetic equation was as follows.

(3) $$\frac{t}{q_{t}}=\frac{1}{k_{2}q_{e}^{2}}+\frac{t}{q_{e}}$$

where, *k*_2_ was the equilibrium rate constant of the pseudo-second-order kinetic model (g·mg^−1^·min^−1^), and *q_e_* and *q_t_* were the BPs adsorption amount (mg·g^−1^) at equilibrium *t* and any time *t* (min), respectively. *k*_2_ could be determined by plotting *t*/*q_t_* versus *t* according to the equation.

Meanwhile, the initial adsorption rate (*h*) equation was as follows:

(4) $$h=k_{2}q_{e}^{2}$$

### 2.5. Adsorption Thermodynamics Analysis

Langmuir and Freundlich models were used to describe the adsorption process [35,36,37].

The Langmuir adsorption isotherm equation was as follows:

(5) $$\frac{C_{e}}{q_{e}}=\frac{1}{bq_{m}^{}}+\frac{C_{e}}{q_{m}}$$

where, *C_e_* was the equilibrium concentration (mg·L^−1^), *q_e_* was the BPs equilibrium adsorption capacity of the adsorbent (mg·L^−1^), *q_m_* was the monolayer adsorption capacity of the adsorbent (mg·L^−1^), *b* was the Langmuir constant related to the affinity of the bending sites and energy of adsorption (L·mg^−1^), and *q_m_* and *b* were obtained from the slope and intercepts of the linear plots of *C_e_*/*q_e_* versus *C_e_*.

The Freundlich adsorption isotherm equation was as follows:

(6) $$\log q_{e}=\log K_{f}+\frac{1}{n}\log C_{e}$$

where, *K_f_* and *n* were the Freundlich adsorption constants which indicated the adsorption capacity and intensity, respectively, and *q*_e_ was the BPs equilibrium capacity of the adsorbent. The model was an empirical equation describing the adsorption onto a heterogeneous surface.

## 3. Results and Discussion

### 3.1. Characterization of the Sample

#### 3.1.1. Characterizations of Biochar and Soil

**Figure 1:**
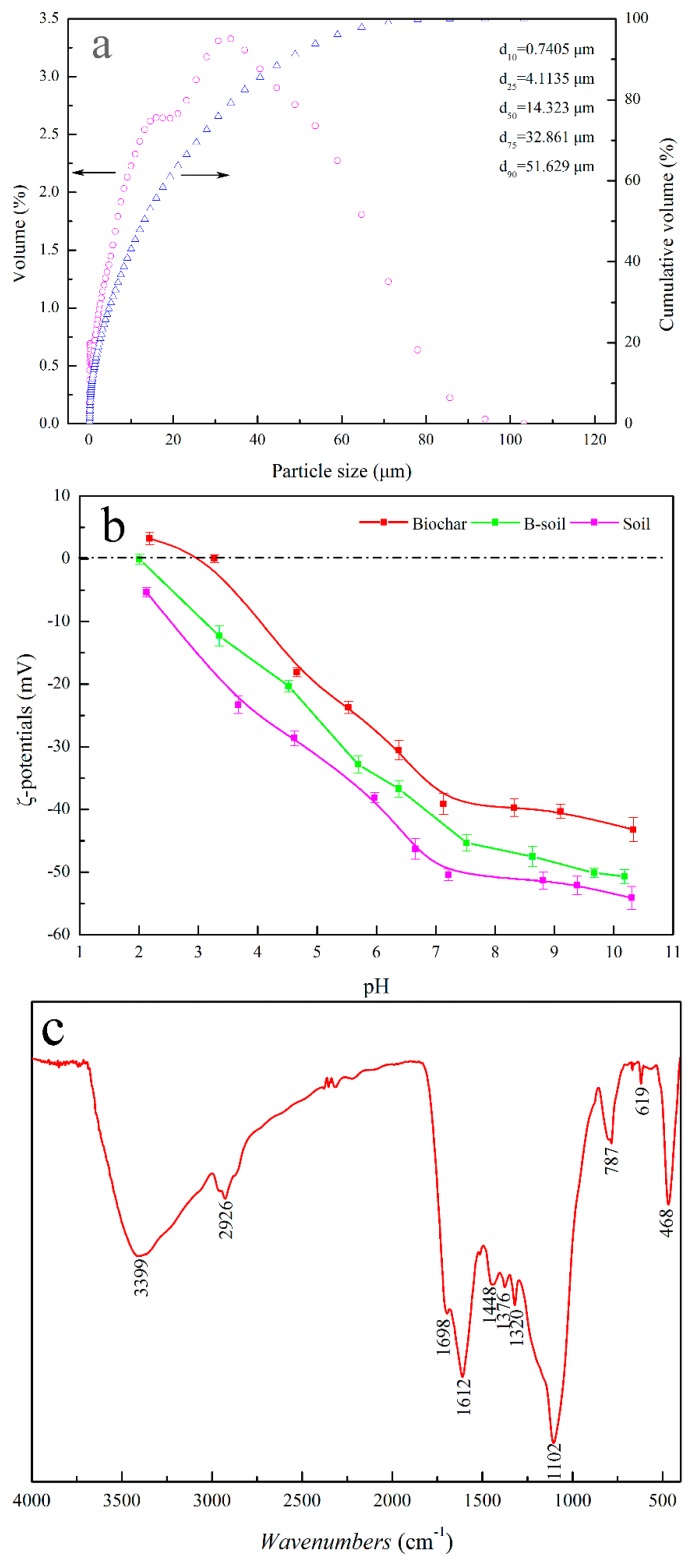
Particle size distribution of the soil sample (**a**), Zeta-potentials of the biochar and soil B-soil samples (**b**), and FT-IR spectra of the biochar sample (**c**).

The physicochemical properties of the biochar and soil are listed in Table S1. The biochar sample shows a relatively high elemental C concentration and low O/C ratio. The H and N contents are relatively low, and the pH value of the biochar sample is alkaline. The specific surface area of the biochar is 204 m^2^∙g^−1^, while the value of the soil sample is only 75 m^2^∙g^−1^. CEC is a measurement of the negative charge of the material surface, which could be neutralized by exchangeable cations [33,38]. The absolute CEC values of biochar, soil, and B-soil are 29.3, 35.7, and 30.2 cmol/kg, respectively. As shown in Figure 1a, the particle size distribution of the soil sample reflects that the soil sample has a broad size distribution, and the *d*_50_ is about 14.323 μm. Figure 1b shows the zeta-potentials of biochar, soil, and B-soil as a function of the pH value, indicating that the points of zero charges (PZCs) of biochar, soil, and B-soil are located at around pH 3.3, <2.0, and 2.0, respectively [25]. The surface functional groups of the biochar are investigated by FT-IR spectra and shown in Figure 1c. The adsorption band near 3400 cm^−1^ is attributed to the stretching vibration of -OH. The bands near 2925 and 2870 cm^−1^ could be attributed to the C-H stretching vibrations of the CH_2_ and -CH_3_ groups, respectively [25], and the bands near 1440 and 1370 cm^−1^ are a result of -CH_2_- scissoring vibrations [39]. The unresolved shoulder peaks at 1700 cm^−1^ and the peak at 1610 cm^−1^ represent the stretching vibration of ester carbonyl groups and the C=O stretching vibrations of amides, respectively [40,41]. The minor peak near 870 cm^−1^ can be attributed to the γ-CH of furan, and the peak near 780 cm^−1^ is related to the β-rings of pyridines [40,42]. The band near 1260 cm^−1^ is probably a result of the stretching vibrations of CO- in aromatic and -OH in phenolic compounds [21], and the bands indicative of C-O-C in aliphatic ethers and -OH in alcohols (1150–1060 cm^−1^) are consistent with the oxygenated functional groups [21,43].

#### 3.1.2. Characterization of DOM

**Figure 2:**
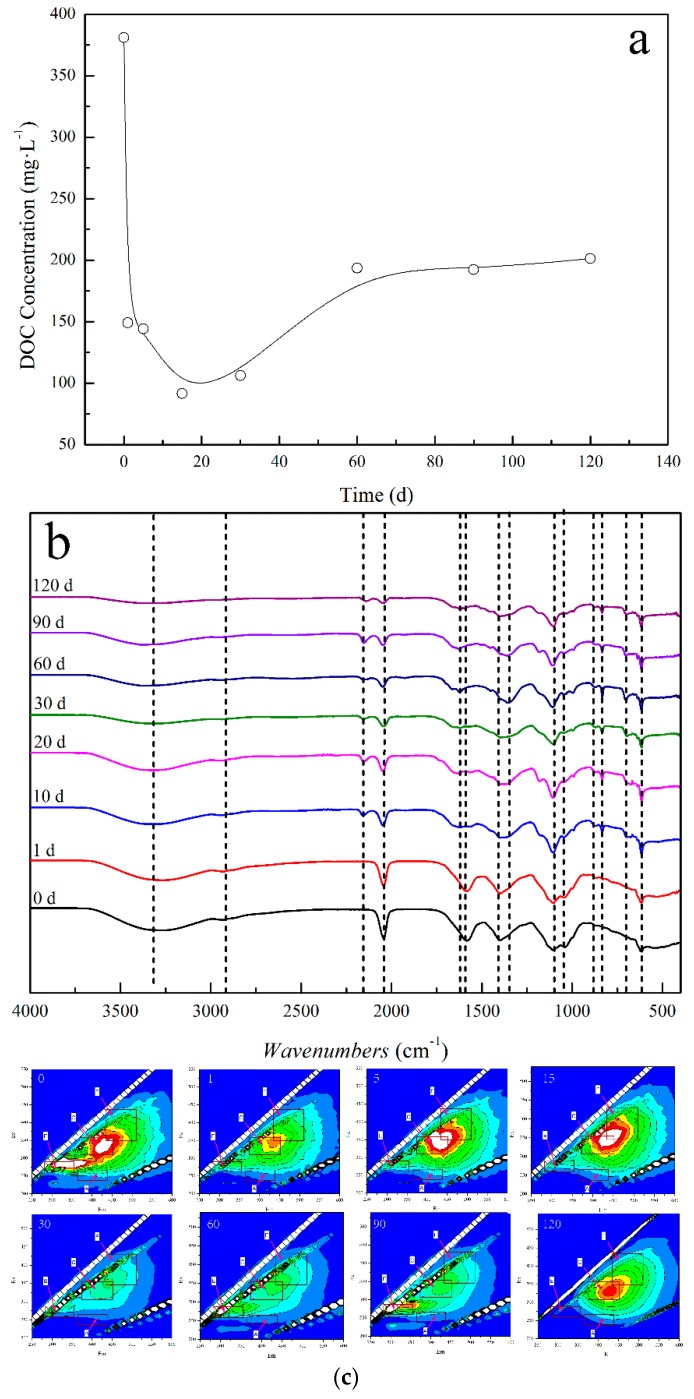
DOC concentration in DOM (**a**), FT-IR spectra of the DOM (**b**), and 3D-EEM spectra of DOM (**c**).

The characterizations of the DOM sample are summarized in Figure 2. In order to investigate the change of the DOM concentration during the decay process, the dissolved organic carbon (DOC) is the common measurement index. As shown in Figure 2a, the concentration of DOM decreased before the first 20 days, then increased, and finally remained stable after 60 days. Moreover, the decrease in the DOM concentration was mainly caused by the utilization of small molecule compounds by microorganisms, while the increase in the concentration may be a result of the conversion of insoluble organic matter to DOM further decomposed by microorganisms. Finally, the utilization of the straw by the microorganisms led to saturation. Hence, the active components of the organic matter in DOM change dramatically during the straw decay process. To investigate the functional groups of DOM, the FT-IR results are shown in Figure 2b. The peak at 3320 cm^−1^ is attributed to the stretching vibration of -OH or -NH in amides. With the extension of the decay, the reduced intensity of this peak illuminates the decreased contents in the COOH, -OH, or -NH. The reduced peak intensities indicate that the aliphatic or cycloaliphatic organic acid are biodegradable, where appear near 2930 and 2870 cm^−1^, attributed to the C–H stretching vibrations of the CH_2_ and -CH_3_ group. Furthermore, the new band near 2150 cm^−1^ demonstrates new chemical bond formation after 10 days. The peak near 2040 cm^−1^ is a result of C≡C asymmetric stretching vibration, which could also be decomposed by the microorganisms. The peak at 1580 cm^−1^ shows the bending vibration of N-H and the stretching vibrations of C=C, C=O, and –COO- in lignin, while the peak shifts to 1640 cm^−1^ and the intensity reduces after 60 days, demonstrating the rapid decomposition of the carboxylic acid lipids or amino acids, or the low decomposition of the lignin. The protein peak near 1400 cm^−1^ shifts to 1350 cm^−1^, indicating the formation of amide compounds. The decreased intensity of the peak near 1035 cm^−1^ is probably a result of the decomposition of sugars. The peak near 615 cm^−1^ can be attributed to the C-H bending vibration of C≡C, and there is no obvious change [22,44]. The fluorescence spectroscopy is an effective method for revealing the change of organic components in DOM. Previous research [45] has shown that the classifications of fluorescent materials and fluorescence peaks in DOM are as follows: The Peak-A at Ex/Em = 240–270/370–440 nm, labeled the fulvic-like compound in the ultraviolet region, is characterized as the components derived from lignin and other degraded plant materials; the Peak-C at Ex/Em = 310–360/370–450 nm is attributed to the fulvic-like compound in the visible region; the Peak-E at Ex/Em = 350–440/430–510 nm is derived from the fluorescence of protein-like substances; and the Peak-F at Ex/Em = 260–290/300–350 nm is ascribed as fluorescence of humic acid-like substances. The 3D-EEM fluorescence spectra of DOM are illustrated in Figure 2c. Peak-A and Peak-F appeared in the initial DOM (0 day), and the other peaks were not outstanding, while Peak-A could be derived from the microorganism parasitized on straw or a trace amount of decomposed substances on the straw surface. With the extension of degradation, Peak-F gradually decreased and finally disappeared, and only Peak-C was left, indicating that the protein in the straw was decomposed by microorganisms and converted into the fulvic-like compounds. Moreover, the ratios of the DOM fluorescence intensity to the total in each area are listed in Table S2. As seen in Table S2, the ratios of *Φ*_peak-A_ and *Φ*_peak-C_ decreased and then increased with the extension of degradation, and the ratio of *Φ*_peak-E_ fluctuated and the overall trend became smaller. The ratio of *Φ*_peak-F_ increased firstly, then decreased quickly, and finally slowly increased. The change of DOM components was mainly caused by the decomposition of microorganisms, which eventually formed deeply decomposed organic matter [46].

### 3.2. Adsorption Results

#### 3.2.1. DOM and BPs Adsorption

**Figure 3:**
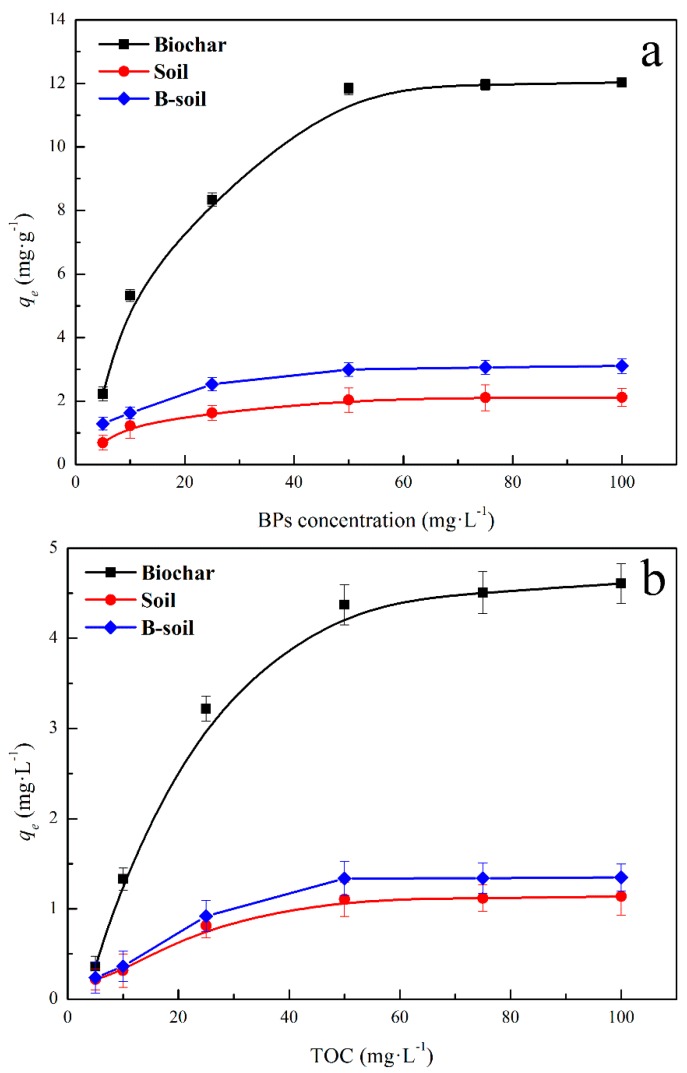
BPs (**a**) and DOM (**b**) equilibrium adsorption amount on the samples.

The BPs adsorption amounts on biochar, soil, and B-soil are shown in Figure 3a, which indicated that the BPs equilibrium adsorption amounts were about 11.36, 2.02, and 2.72 mg·g^−1^, respectively. The biochar had a large BPs adsorption amount and could improve the BPs adsorption capacity of the soil. The difference in the equilibrium adsorption amount was mainly due to the specific surface area, pore structure, or functional groups. In order to explore the DOM adsorption behavior on the samples, the results were presented as shown in Figure 3b. DOM adsorption amounts on biochar, soil, and B-soil exhibited a positive correlation with the initial DOM concentration and the amounts then eventually stabilized (about 4.51, 1.11, and 1.34 mg·g^−1^, respectively). Briefly, the BPs adsorption amounts of B-soil increased by 30% compared to soil, indicating that the biochar could improve the adsorption capacity of the soil. For DOM adsorption (Figure 3b), biochar also exhibited a better adsorption capacity than the soil. Both DOM and BPs could be adsorbed on the soil and biochar surface.

#### 3.2.2. Effect of DOM with Different Decomposition Times

**Figure 4:**
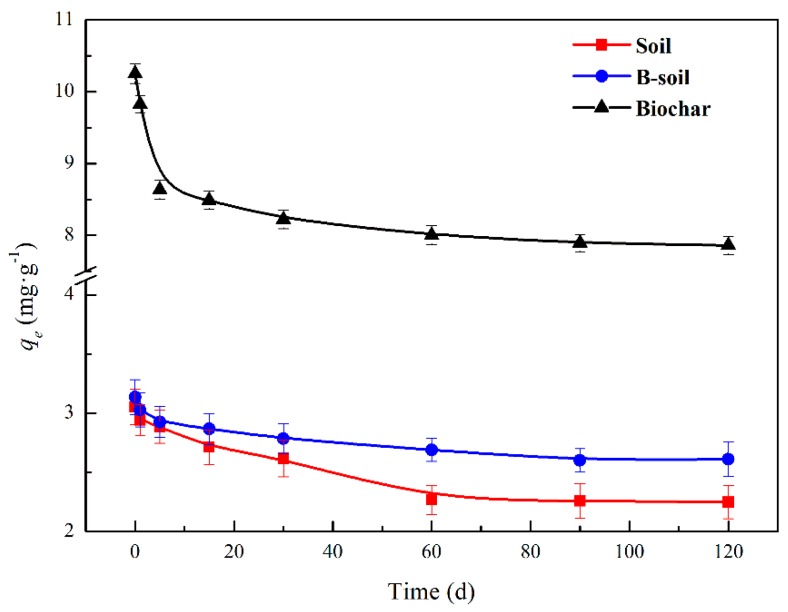
Effect of DOM with different degrees of decomposition on BPs adsorption on the samples.

The components and functional groups of DOM with different degrees of decomposition were significantly different, which is shown in Section 3.1.2. The effect of DOM with different degrees of decomposition on the BPs adsorption on the samples is shown in Figure 4. Compared to the results of Figure 3a, DOM displayed an improvement in the BPs adsorption on the soil and an inhibition to that on B-soil and biochar, and the trends of the three adsorbents were similar. With the extension of straw decomposition, the effect of DOM on the BPs adsorption became weak, and it tended to be stable after 60 days. The reason for the increase in the BPs adsorption on soil could be due to the co-adsorption between DOM and BPs on the soil surface, while the decrease in the adsorption on B-soil and biochar may be due to the competitive adsorption between them. The phenomenon may be caused by the differences in surface properties of soil and biochar [47,48]. Considering the tendency, it may be related to the change of the components and functional groups of DOM. In the initial stage, the main substances in DOM were hydrophilia, as carbohydrates, amides, and aliphatics. As degradation progressed, DOM was degraded into a mass of small molecular acids or intermediates by microorganisms. In the later period, the hydrophobicity of DOM was enhanced, which was related to the aromaticity of DOM increasing and consistent with the results of DOM characterizations [22,45,46]. During the process, the hydrophobicity of DOM gradually increased, and BPs was more soluble in DOM, resulting in a decrease in the amount of BPs adsorbed.

#### 3.2.3. Adsorption Kinetics

**Figure 5:**
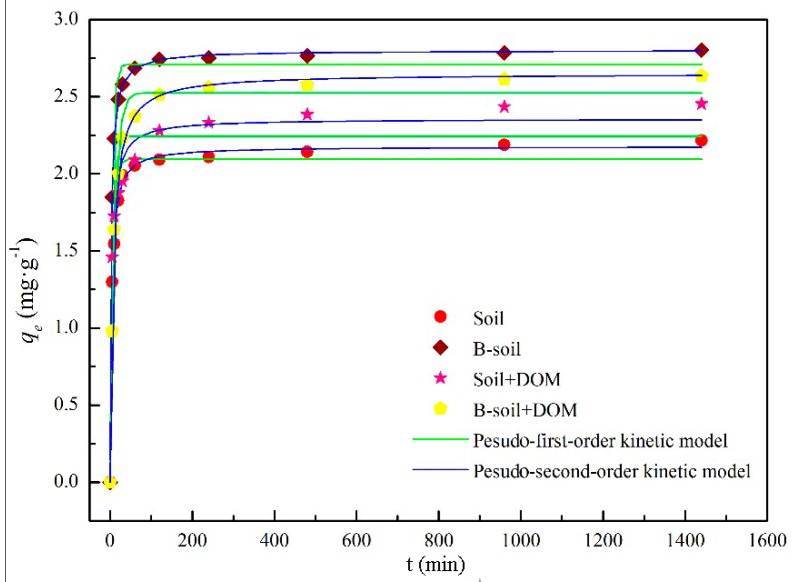
Relation between BPs adsorption amount and the contact time.

Figure 5 and Figure S2 show that all adsorption kinetic data from the soil and B-soil samples with or without DOM could be described very well by the pseudo-second-order kinetic model with higher correlation coefficients (*R*^2^), indicating that BPs adsorption of soil and B-soil samples is always a chemical process, irrespective of DOM [25,49]. In addition, the equilibrium adsorption times were 90 and 120 min for soil and B-soil without DOM, while the equilibrium times were 120 and 240 min with DOM, respectively. The extended time could be caused by the interaction between DOM and BPs. Meanwhile, the adsorption process was divided into two portions, as rapid adsorption and slow adsorption. The rapid adsorption process could be accomplished quickly, while the slow adsorption process was the saturated adsorption and slow. The fitting parameters of the pseudo-second-order kinetic model are listed in Table 1. As listed in the table, the calculated *q_e_* values were approximately consistent with the experimental data. In terms of the *h* values, the rate for the B-soil sample was greater than that of the soil sample. In terms of adsorption constants (*k*_2_), the larger the *k*_2_ value, the slower the adsorption rate [50], and the variation tendency was contrary to the changes in the *h* values, irrespective of DOM.

#### 3.2.4. Adsorption Isotherms

**Figure 6:**
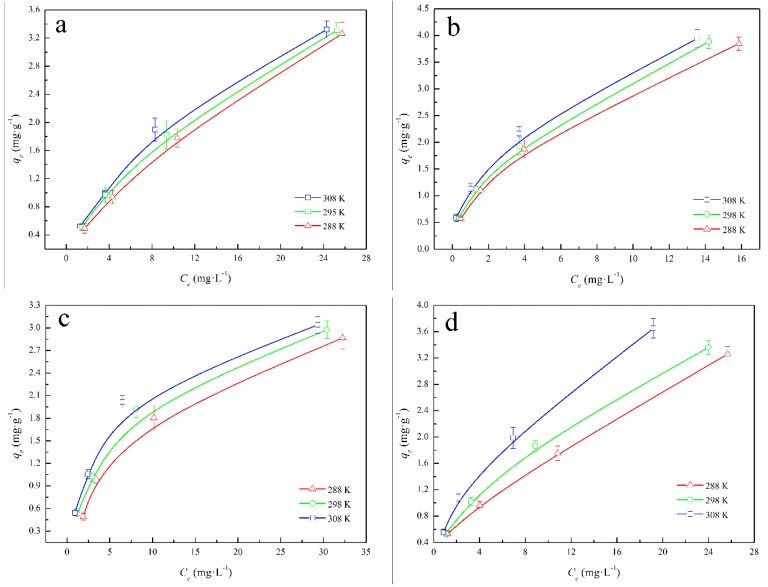
Adsorption isotherms of soil (**a**), B-soil (**b**), soil+DOM (**c**), and B-soil+DOM (**d**).

The equilibrium adsorption isotherm is fundamental to describing the interactive behavior between solutes and sorbents and important for the design of an adsorption system. Figure 6a,b show the BPs adsorption capacities of soil and B-soil at different initial BPs concentrations and adsorption temperatures, and Figure 6c,d show the adsorption isotherms of soil and B-soil with DOM. It is evident that the initial BPs concentrations and adsorption temperatures affect the adsorption capacity of BPs for all samples: adsorption capacities increase as the initial concentrations increase, and favorable adsorption occurs at a higher temperature, irrespective of the DOM. The fitting results of the Langmuir model and the Freundlich model are listed in Table 2. The isotherm constants and the correlation coefficients (*R*^2^) were obtained by linear regression and indicate that the adsorption processes could be well-described by the Freundlich model ($R_{Freundlich}^{2}$ > $R_{Langmuir}^{2}$) for soil and B-soil samples, which reveals that the BPs is adsorbed in multiple layers on the sample surfaces [51,52,53]. Meanwhile, the Freundlich model was also fitted to the BPs adsorption with DOM. In the fitting results of the Freundlich model, the *1/n* values were all below 0.5, indicating that the BPs could easily be adsorbed on the soil and B-soil sample surfaces [33,50]. The *K_f_* values represent the adsorption capacity of the adsorbent [25], and the B-soil samples showed higher *K_f_* values than the other samples, irrespective of the temperature. The thermodynamic parameters for the BPs adsorption are listed in Table 3. Δ*S*^0^ and Δ*H*^0^ were calculated from the slope and intercept of *Van’t Hoff* plots of ln*K_d_* versus *1/T*. In Table 3, negative Δ*G*^0^ and positive Δ*H*^0^ indicate that the BPs adsorption process is spontaneous and endothermic, and the greater the absolute Δ*G*^0^ value, the more spontaneous the adsorption. The positive Δ*S*^0^ reflects an increase in randomness at the solid/solution interface during BPs adsorption of the samples. On the other hand, the Δ*H*^0^ values of soil samples at different initial BPs concentrations are less than those of B-soil samples. However, after injecting DOM, the Δ*H*^0^ values of soil samples at different initial BPs concentrations are greater than those of soil samples without DOM, while the Δ*H*^0^ values of B-soil samples are less than the values of B-soil samples without DOM. The absolute Δ*G*^0^ values of all the experiments increased with the temperature increase, indicating that the adsorption behavior was easily more spontaneous at a high temperature. Additionally, the absolute Δ*G*^0^ values of the soil sample with DOM were greater than the absolute values of the soil without DOM, and the absolute values of the B-sample with DOM were less than those of the B-sample without DOM.

### 3.3. Instructional Application

**Figure 7:**
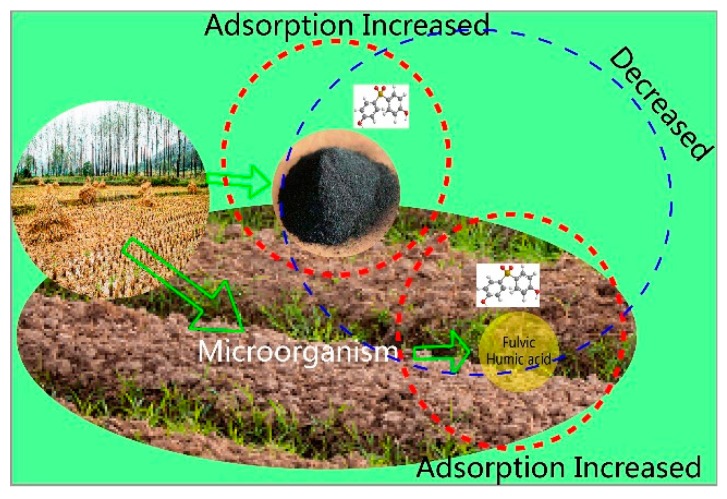
The interaction between soil and DOM.

In recent years, straw-returning to the field has been widely promoted as one of the most important methods for reducing the pollution caused by straw burning, and it could also increase the organic matter of soil. The proposed adsorption model was shown in Figure 7. The straw could be degenerated into DOM released into the soil, and DOM plays an important role in the natural environment as a microreactor of contaminants. Furthermore, biochar has developed into an important soil amendment. However, there is an inconsistent discussion about the interaction between biochar and DOM on the contaminants. The results of the experiments show that both DOM and biochar could improve the BPs adsorption capacity of soil, while there is a decrease in the BPs adsorption on biochar modified soil samples with DOM.

It could be seen that straw-returning to the field would enhance the BPs adsorption capacity of the soil sample, while it would reduce the adsorption capacity of biochar modified soil. The conclusions may contribute to the comprehensive utilization of straw and farm land protection, and for BPs, it has been shown that only the addition of biochar or straw can promote the BPs adsorption capacity of soil, but they cannot be applied at the same time. This is important for the migration of contaminants in the soil.

## 4. Conclusions

This paper investigated the BPs adsorption behavior of red acid soil and biochar modified soil (B-soil) with DOM. The soil sample was collected in the south of China, and DOM was decayed and extracted from the soil sample for a certain time using the paddy straw as the feedstock. With the extension of decay, the aromaticity of DOM increased. The adsorption tests indicated that both DOM and biochar could improve the BPs adsorption capacity of soil, while DOM could decrease the BPs adsorption on biochar. Furthermore, the BPs adsorption capacities increased with the initial BPs concentrations increase, and the capacities also increased with the adsorption temperature increase. The conclusion may contribute to the migration of contaminants in the soil, and for BPs, only the addition of biochar or straw can promote the BPs adsorption capacity of soil, but they cannot be applied at the same time.

## Figures and Tables

**Table 1:** Fitting parameters of the pseudo-second-order kinetic model.

Sample	*k*_2_ (g·mg^−1^·min^−1^)	*h* (mg·g^−1^·min^−1^)	*q_e_* (mg·g^−1^)	*R* ^2^
Soil	0.0246	0.1300	2.2999	0.9969
B-soil	0.0170	0.1430	2.9011	0.9995
Soil + DOM	0.0513	0.3104	2.4594	0.9999
B-soil + DOM	0.0511	0.3571	2.6427	0.9999

**Table 2:** Fitting results of the Freundlich model.

Sample	T (K)	*R* ^2^	*K_f_*	1/n	Sample	T (K)	*R* ^2^	*K_f_*	1/n
Soil	288	0.9996	0.5383	0.3411	Soil + DOM	288	0.9955	0.5837	0.3293
	298	0.9980	0.5580	0.4061		298	0.9929	0.6081	0.4036
	308	0.9919	0.5992	0.4512		308	0.9964	0.6174	0.4397
B-soil	288	0.9996	0.6412	0.3037	B-soil + DOM	288	0.9901	0.6032	0.3181
	298	0.9994	0.6556	0.3562		298	0.9999	0.6298	0.3346
	308	0.9990	0.6959	0.3815		308	0.9946	0.6712	0.4040

**Table 3:** Thermodynamic parameters for adsorption of BPs on the samples.

Sample	*C*_0_ (mg·L^−1^)	Δ*H*^0^ (kJ·mol^−1^)	Δ*S*^0^ (J·mol^−1^·K^−1^)	Δ*G*^0^ (kJ·mol^−1^)		
				308 K	298 K	288 K
Soil	10	20.5152	114.4782	−24.80672	−23.46912	−22.13144
	20	12.0577	107.5530	−24.21392	−23.11512	−22.01632
	40	10.7049	80.7331	−21.56264	−20.58496	−19.60728
	80	7.9711	86.6679	−20.58432	−19.70936	−18.83448
B-soil	10	28.8197	184.6734	−48.591202	−45.396238	−35.510153
	20	17.0630	139.3851	−44.787105	−42.375831	−32.576592
	40	13.2305	105.3929	−41.946099	−40.122852	−30.384682
	80	10.8852	97.8035	−40.227517	−38.535577	−29.123512
Soil + DOM	10	23.8728	133.7623	−27.84576	−26.01408	−24.1824
	20	15.6082	109.8818	−26.45488	−24.73392	−23.01312
	40	11.0857	97.7659	−28.49872	−26.884	−25.26928
	80	8.8361	87.4938	−26.99296	−25.6064	−24.21968
B-soil+DOM	10	25.1692	166.2061	−39.32236	−36.73684	−28.73654
	20	116.2261	125.4466	−36.2439	−34.29258	−26.36256
	40	12.3845	100.1233	−33.94482	−32.46936	−24.58876
	80	9.5069	88.0232	−32.55406	−31.18486	−23.56816

## Supplementary Materials

The following are available online at https://www.mdpi.com/1660-4601/16/5/764/s1, Figure S1: Molecular structures of BPa and BPs, Figure S2: The pseudo-second-order kinetic fitting line, Table S1: Physicochemical properties of the soil sample and biochar sample, Table S2: The ratio of fluorescence intensity in each region to total fluorescence intensity of DOM.
