# Supplementary material for: Bisphenol S Adsorption Behavior on Ferralsol and Biochar Modified Soil with Dissolved Organic Matter
Source: Int J Environ Res Public Health. 2019 Mar 3;16(5):764. doi: 10.3390/ijerph16050764 (PMC6427365; doi:10.3390/ijerph16050764)
Supplement: Supplementary file 1 [file ijerph-16-00764-s001.zip › ijerph-433410-suppl xml.pdf]

1. Physicochemical Analyses

pH: Biochar was addition in de-ionized water with a 1:10 (*m*:*V*) suspension under stirring for 1 h using an electromagnetic stirrer, the pH values of the mixture were executed using the Sartorius PB-10 digital pH meter (Germany).

Zeta-potential: The Zeta-potential of the biochar samples were performance at the temperature of 25 °C by a Zetasizer Nano (Zs90, Malvern Instruments, UK) (Wang et al., 2017). During the measurement, the suspension was monitored continuously in aspect of the conductivity and pH value. The average of three times zeta potential measurements were reported as the results.

Table S1. Physicochemical properties of the soil sample and biochar sample.

Sample	SA ($m^2 \cdot g^{-1}$)	C (%)	O (%)	N (%)	S (%)	H/C	O/C	pH	CEC
Soil	75	2.48	9.11	0.17	0.58	4.39	3.37	6.28	35.7
Biochar	204	60.02	29.27	0.58	0.25	1.03	0.37	7.21	29.3

Table S2. The ratio of fluorescence intensity in each region to total fluorescence intensity of DOM.

Time (d)	A (%)	C (%)	E (%)	F (%)
0	0.63	3.36	1.31	9.42
1	0.54	3.57	0.81	10.56
5	0.45	3.41	0.55	11.29
15	0.49	3.63	0.50	12.02
30	0.28	2.15	0.88	6.78
60	0.26	1.51	0.83	7.50
90	0.32	1.57	1.02	8.63
120	1.36	11.00	0.46	12.96

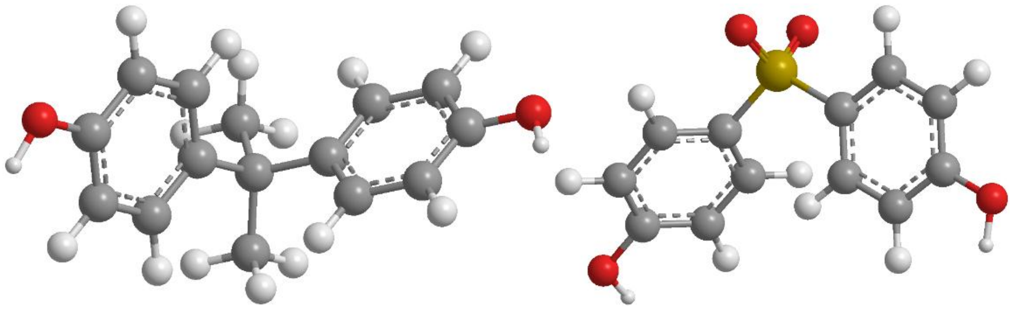

Figure S1. Molecular structures of BPa and BPs.

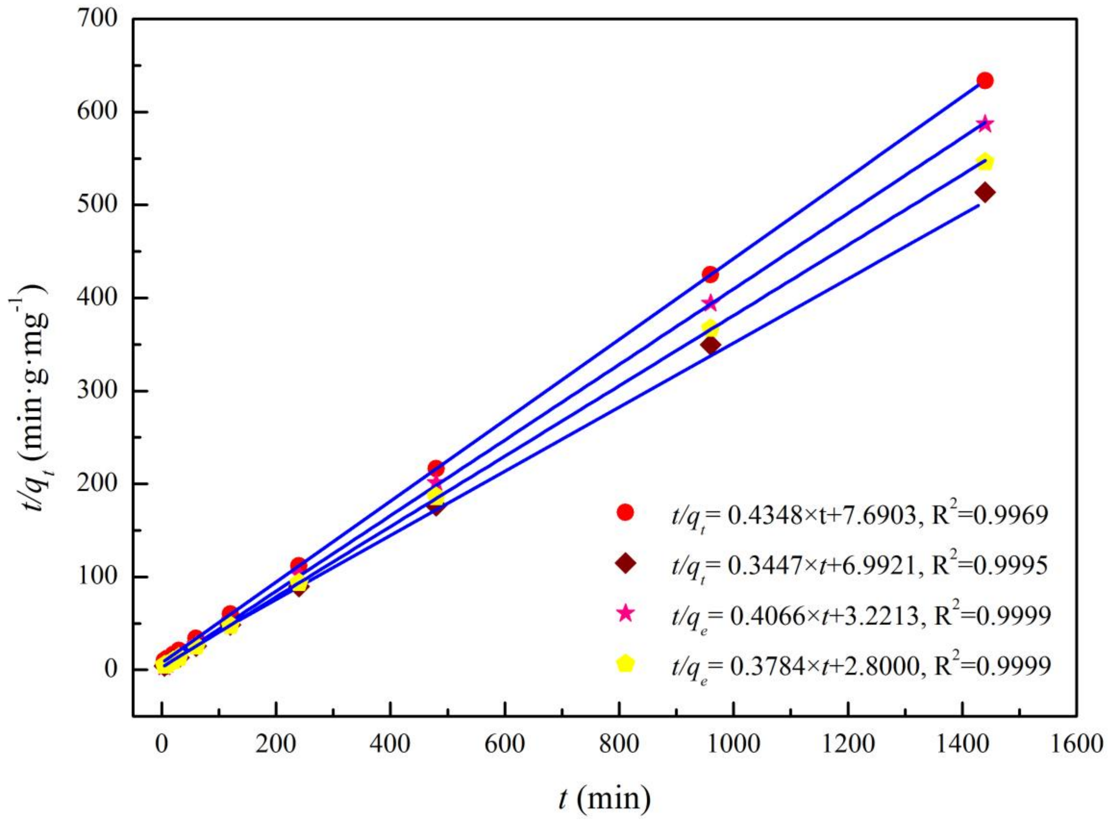

Figure S2. The pseudo-second-order kinetic fitting line.
